# Stepwise rescue management of cataclysmic postpartum spontaneous coronary artery dissection using multimodal imaging

**DOI:** 10.1093/ehjcr/ytaf613

**Published:** 2025-11-30

**Authors:** Jérémie Macia, Jean-Christophe Macia, Alain Tavildari, Pascal Motreff

**Affiliations:** Department of Cardiology, Gabriel-Montpied Hospital, University Hospital of Clermont-Ferrand, CHU Gabriel-Montpied 58 rue Montalemberg, Clermont-Ferrand 63000, France; Department of Cardiology, Arnaud de Villeneuve Hospital, University Hospital of Montpellier, 371 Av. du Doyen Gaston Giraud, 34090 Montpellier, France; Department of Cardiology, Valais Romand Hospital, Av. du Grand-Champsec 86, 1950 Sion, Switzerland; Department of Cardiology, Gabriel-Montpied Hospital, University Hospital of Clermont-Ferrand, CHU Gabriel-Montpied 58 rue Montalemberg, Clermont-Ferrand 63000, France

A 34-year-old woman with no prior history or risk factors for cardiovascular disease was admitted four days postpartum with severe chest pain. While spontaneous coronary artery dissection (SCAD) was suspected, a coronary computed tomography angiography was interpreted as normal and ruled out the diagnosis of aortic dissection. Although transthoracic echocardiogram was normal, recurrent chest pain, elevated troponin levels and electrocardiographic abnormalities prompted urgent invasive coronary angiography (ICA) (*Figure 1*).

**Figure 1:**
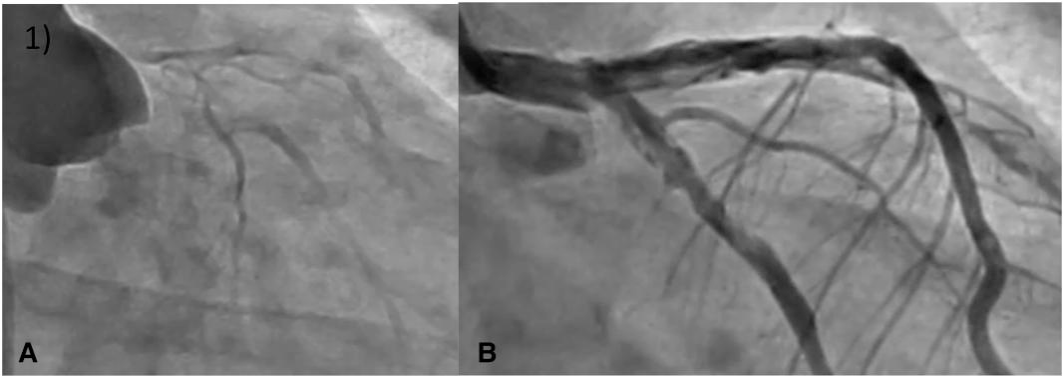
Initial coronary angiogram. (*A*) aortography of the LCA with a TIMI 1 flow. (*B*) ICA of the LCA after implantation of ECLS.

During a period of haemodynamic instability complicated by cardiac arrest, the diagnosis was confirmed via aortography showing a compressive haematoma in the left coronary artery (LCA) with a TIMI 1 flow. A conservative, non-interventional approach was adopted, with extracorporeal life support (ECLS), resulting in rapid stabilization.^1^ Invasive coronary angiography (ICA) visualized an extensive SCAD and highlighted the anticipated challenges of emergency revascularization. ECLS was successfully weaned after five days.

Despite occurrence of bilateral vertebral artery dissection, discharge was possible one month later without cardiac or neurological sequelae. Follow-up with non-invasive imaging demonstrated regression of the vertebral artery dissection and stability of the coronary lesion.

At 6-month, patient developed exertional angina. CCTA revealed a persistent dissection with aneurysmal changes involving the left anterior descending artery (LAD) (*Figure 2*). CCTA offered detailed anatomical insights into the SCAD (juxta-ostial intimal flap, false lumen positioned above the true lumen, full extent of the dissection)—critical for planning the interventional strategy.^2^ The procedure was guided in real time using optical coherence tomography (OCT), ensuring precise stent placement and enhanced procedural safety (lumen analysis, intimal tears identification, guidewires positioning) (*Figures 3* and *4*), and optimizing the cautious percutaneous coronary intervention (PCI) : Direct stenting (Boston® SYNERGY 3.5 × 48 mm) from left main artery (LM) to LAD (minimal ballooning to avoid dissection extension), Proximal optimization technique (POT)-side (towards circumflex artery)-POT technique and final OCT control confirming closure of false lumen and correct stent expansion (*Figure 5*).^3^

**Figure 2:**
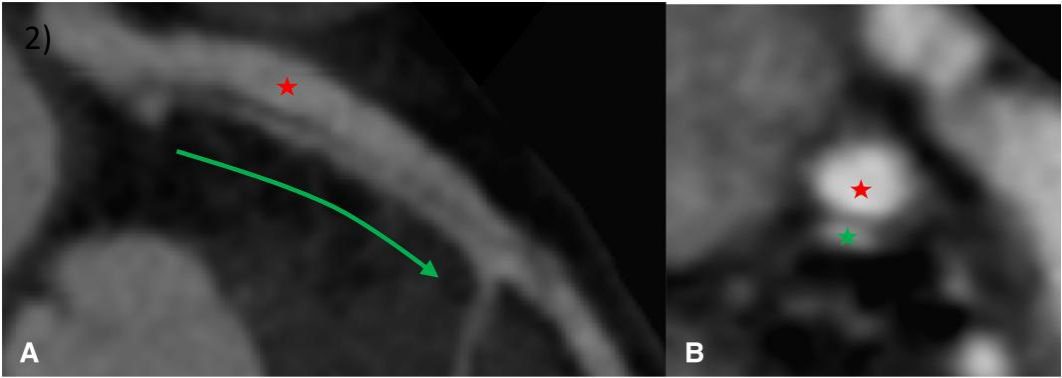
Coronary CCTA at 6 months. (*A*) aneurysmal evolution (*) of the LCA dissection towards the LAD. (*B*) the true lumen (*) compressed by the false lumen (*).

**Figure 3:**
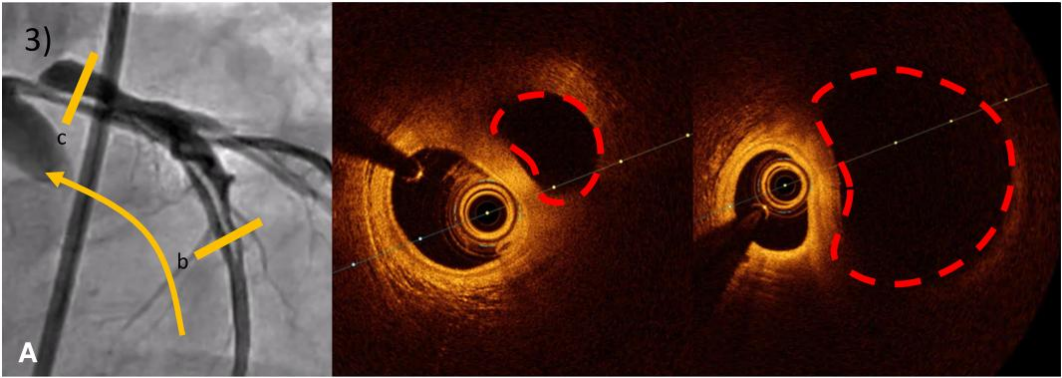
ICA with OCT analysis. (*A*) angiography of LCA’s aneurysm (OCT pullback direction represented by the yellow line). (b/c) OCT of LCA’s aneurysm (false lumen in dotted line).

**Figure 4:**
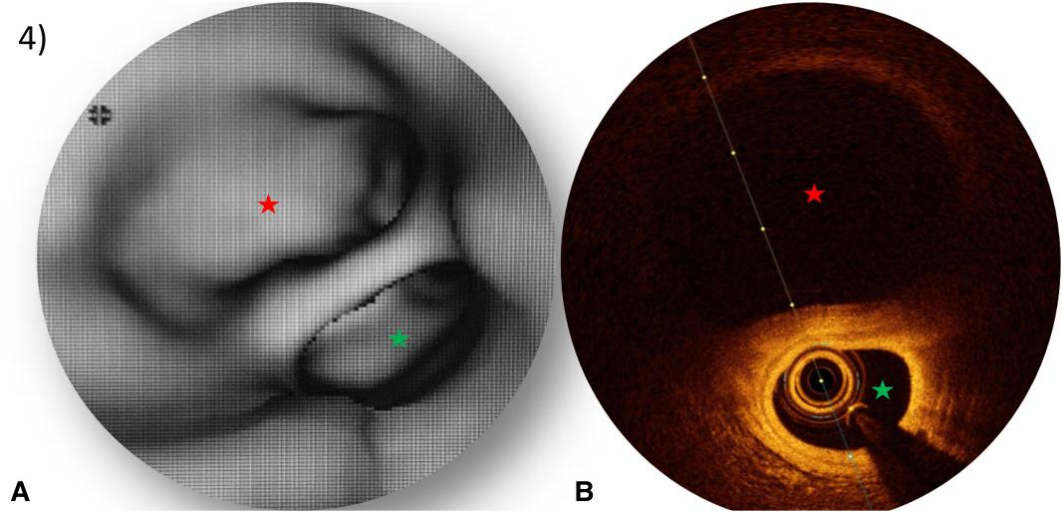
Comparison between CCTA and OCT analysis of the aneurysm. (*A*) Intra-luminal navigation showing the false lumen (*) and the true lumen (*). (*B*) OCT of LCA’s aneurysm (*).

**Figure 5:**
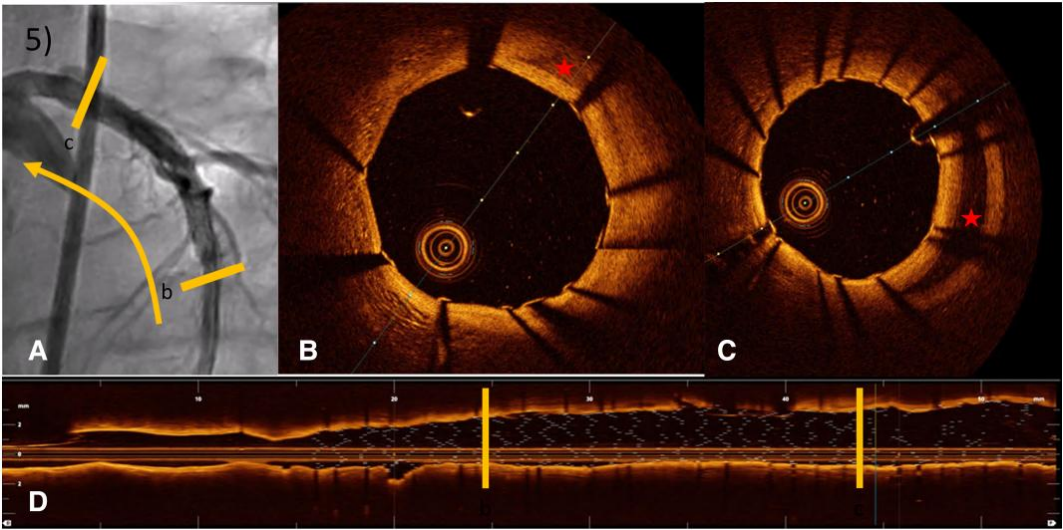
OCT images of LCA PCI in the direction of the LAD. (*A*) ICA with OCT catheter. (*B*) OCT ensuring good expansion of the stent in true lumen. (*C*) OCT confirming coverage of the dissection with the displaced false lumen (*). (*D*) Longview OCT with visualization of the stent.

The left ventricular ejection fraction (LVEF) at discharge was 53%.

After 6 months, CCTA confirmed SCAD healing. At 2 years follow-up, patient remained asymptomatic.

## Supplementary Material

ytaf613_Supplementary_Data

## Data Availability

The data underlying this article will be shared on reasonable request to the corresponding author.
