# Supplementary figures and images for: Stepwise rescue management of cataclysmic postpartum spontaneous coronary artery dissection using multimodal imaging
Source: Eur Heart J Case Rep. 2025 Nov 30;9(12):ytaf613. doi: 10.1093/ehjcr/ytaf613 (PMC12723234; doi:10.1093/ehjcr/ytaf613)

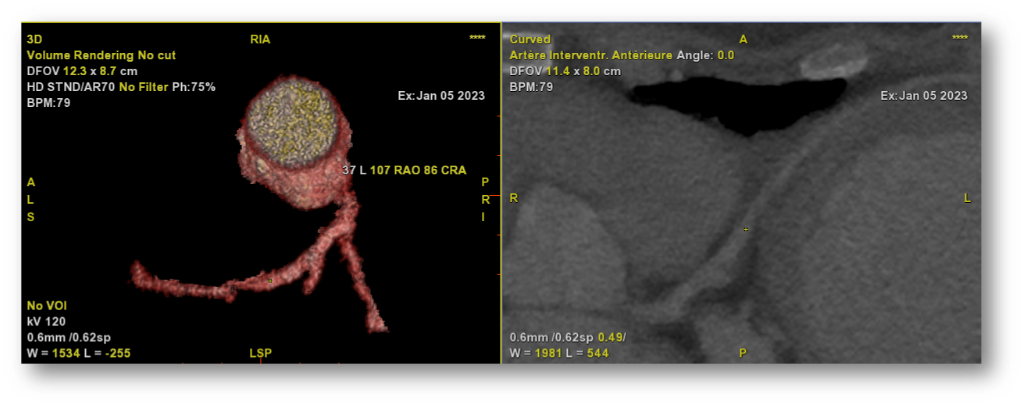

Supplement: ytaf613_Supplementary_Data [file ytaf613_supplementary_data.zip › CCTA M4.png]

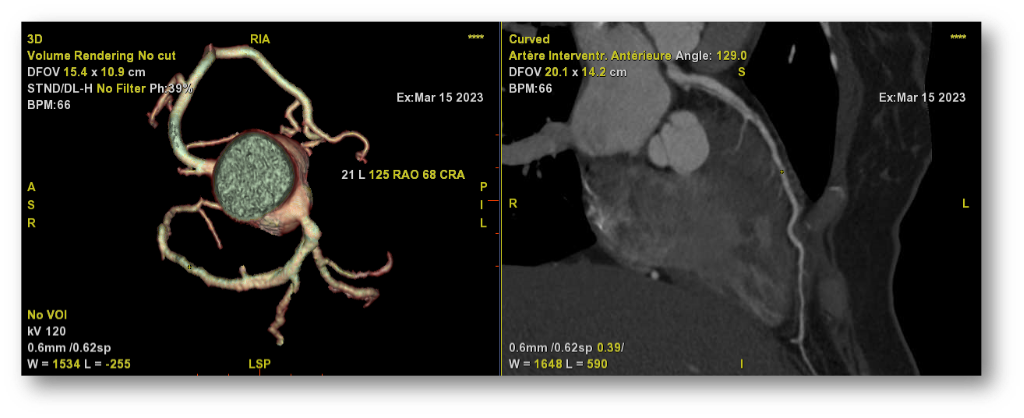

Supplement: ytaf613_Supplementary_Data [file ytaf613_supplementary_data.zip › CCTA M6 1.png]

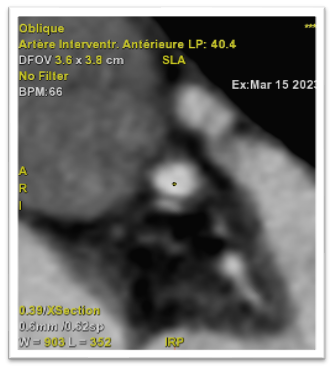

Supplement: ytaf613_Supplementary_Data [file ytaf613_supplementary_data.zip › CCTA M6 2.png]

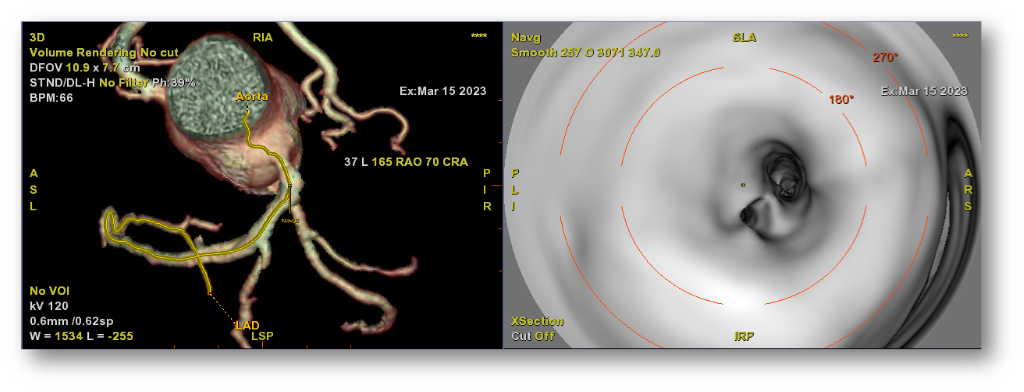

Supplement: ytaf613_Supplementary_Data [file ytaf613_supplementary_data.zip › CCTA M6 3.png]

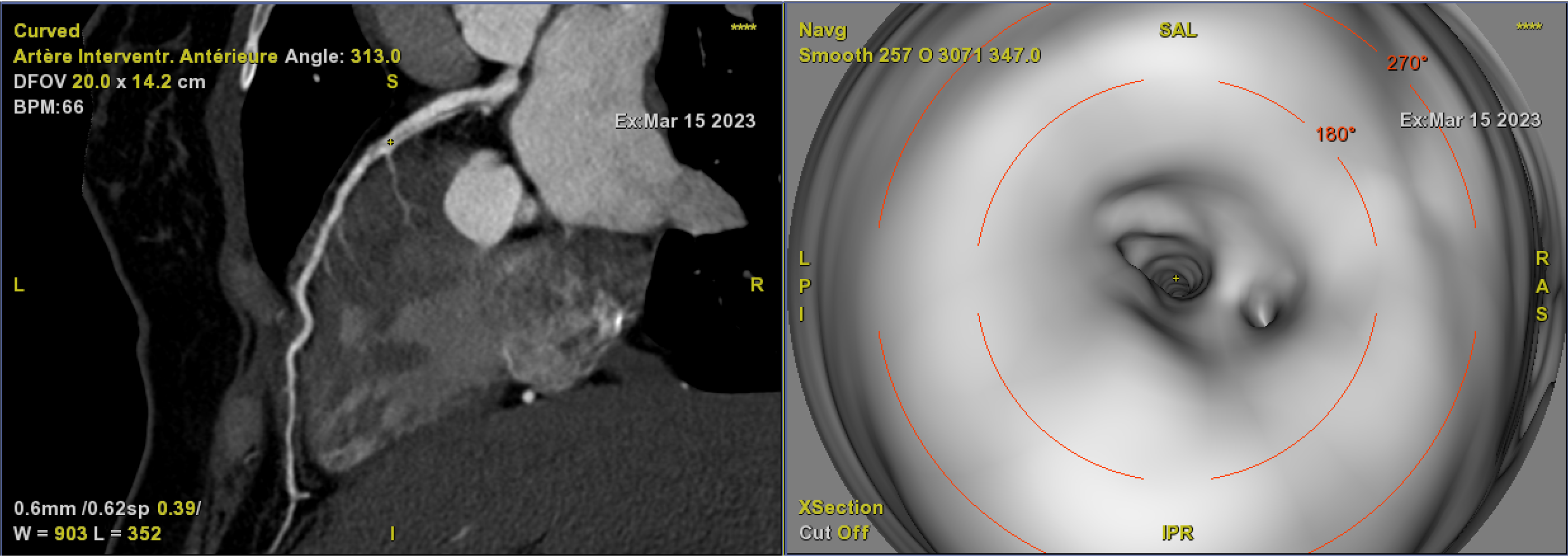

Supplement: ytaf613_Supplementary_Data [file ytaf613_supplementary_data.zip › CCTA M6 4.png]

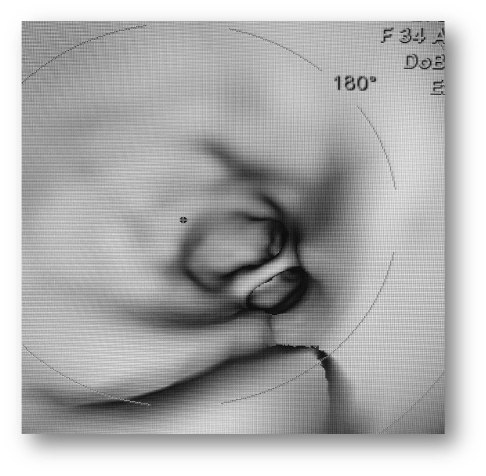

Supplement: ytaf613_Supplementary_Data [file ytaf613_supplementary_data.zip › CCTA M6 5.png]

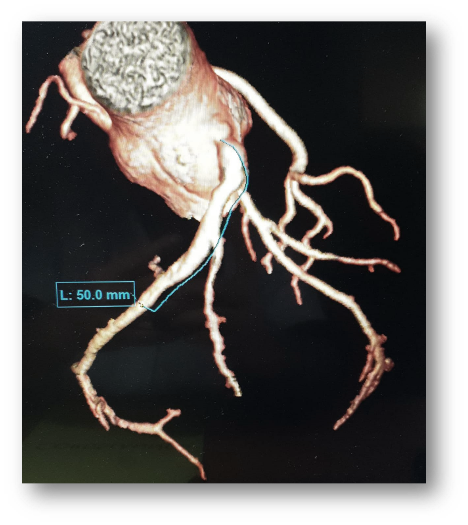

Supplement: ytaf613_Supplementary_Data [file ytaf613_supplementary_data.zip › CCTA M6 6.png]

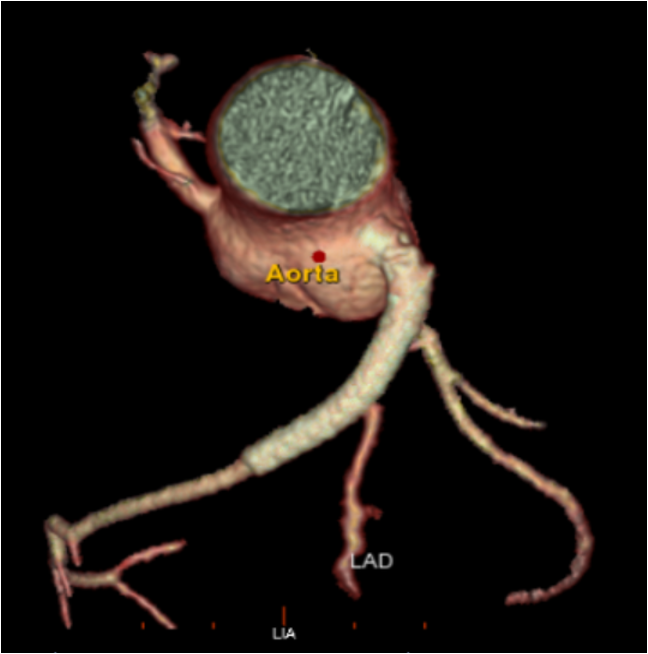

Supplement: ytaf613_Supplementary_Data [file ytaf613_supplementary_data.zip › CCTA M6 after PCI 1.png]

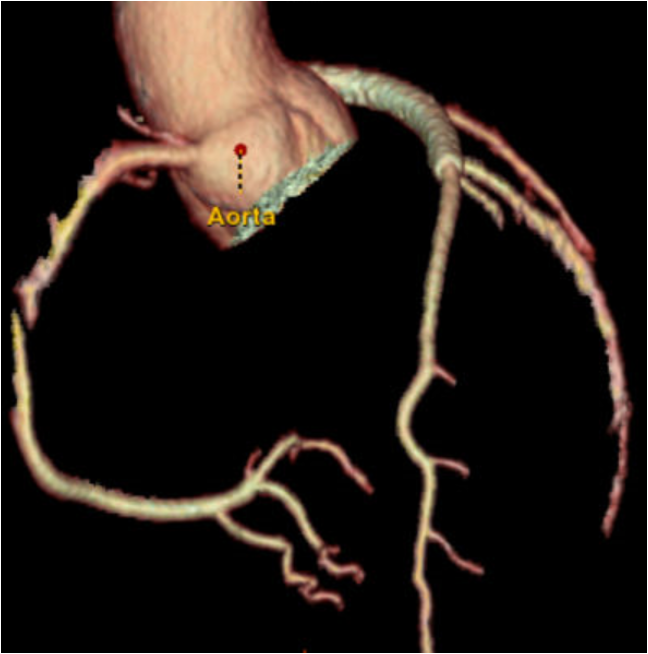

Supplement: ytaf613_Supplementary_Data [file ytaf613_supplementary_data.zip › CCTA M6 after PCI 2.png]

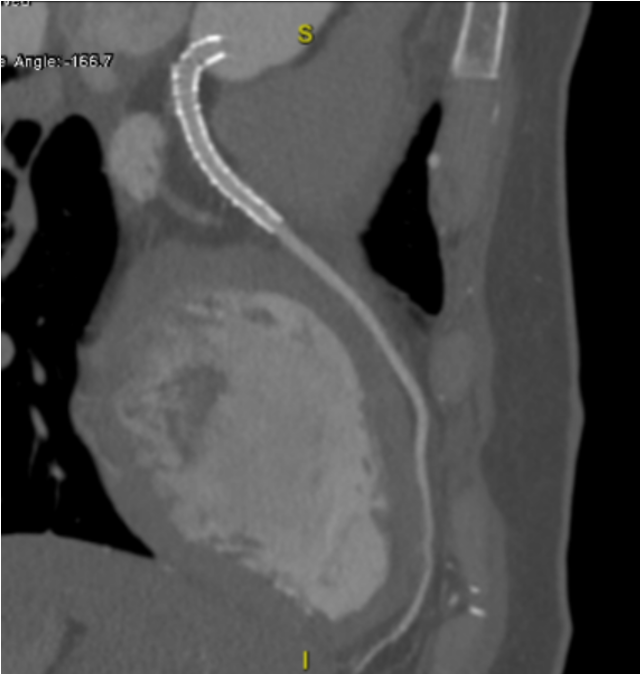

Supplement: ytaf613_Supplementary_Data [file ytaf613_supplementary_data.zip › CCTA M6 after PCI 3.png]

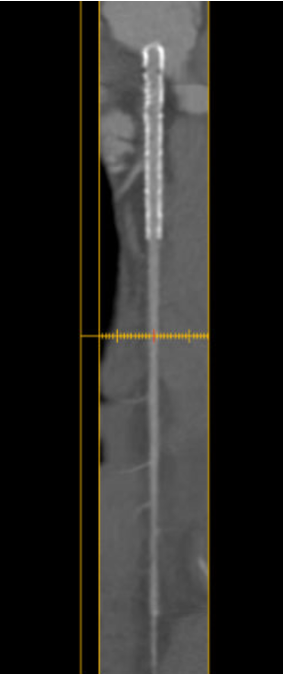

Supplement: ytaf613_Supplementary_Data [file ytaf613_supplementary_data.zip › CCTA M6 after PCI 4.png]

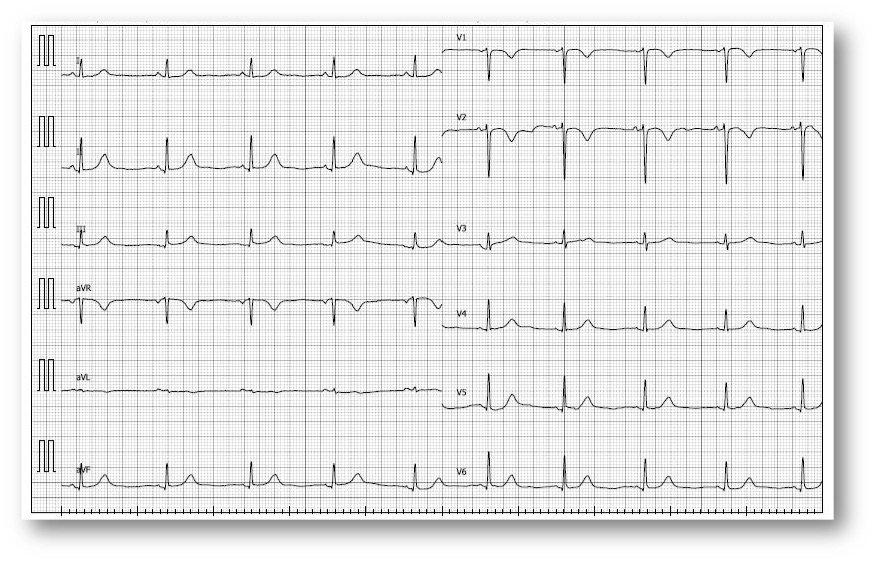

Supplement: ytaf613_Supplementary_Data [file ytaf613_supplementary_data.zip › EKG.jpg]
